# Facile synthesis of graphene-tin oxide nanocomposite derived from agricultural waste for enhanced antibacterial activity against *Pseudomonas aeruginosa*

**DOI:** 10.1038/s41598-019-40916-9

**Published:** 2019-03-12

**Authors:** Anu N. Mohan, Manoj B, Sandhya Panicker

**Affiliations:** 1 Materials Science Research Laboratory, Department of Physics and Electronics, CHRIST (Deemed to be University), Bengaluru, 560029 Karnataka India; 2 Department of Botany, St. Joseph’s College, Post Graduate and Research Center, Langford Road, Bengaluru, 560027 Karnataka India

## Abstract

Antibacterial screening of graphene-tin oxide nanocomposites synthesized from carbonized wood and coconut shell is investigated against *Pseudomonas aeruginosa* for the first time. Efficient and facile one step hydrothermal process adopted in the present work for the synthesis of graphene-tin oxide nanoparticles provides an ideal method for the economic large-scale production of the same. Graphene-tin oxide nanocomposites derived from wood charcoal possess a spherical morphology whereas rod like structures are seen in the case of coconut shell derivatives. An excitation independent fluorescence response is observed in graphene-tin oxide nanohybrids while graphene oxide nanostructures exhibited an excitation dependent behavior. These hydrophilic nanostructures are highly stable and exhibited no sign of luminescence quenching or particle aggregation even after a storage of 30 months. Bactericidal effects of the nanostructures obtained from coconut shell is found to be relatively higher compared to those procured from wood. This variation in antibacterial performance of the samples is directly related to their morphological difference which in turn is heavily influenced by the precursor material used. MIC assay revealed that coconut shell derived graphene-tin oxide composite is able to inhibit the bacterial growth at a lower concentration (250 μg/mL) than the other nanostructures. Nanocomposites synthesized from agro-waste displayed significantly higher antimicrobial activity compared to the precursor and graphene oxide nanostructures thereby making them excellent candidates for various bactericidal applications such as disinfectants, sanitary agents etc.

## Introduction

Recently, quest for the bulk production of graphene based biocompatible materials has gained momentum owing to their wide range of applications as biosensors, purification membranes, sanitary agents etc^1–6^. One of the biggest challenges in the modern era medicine and sanitation industry is the containment of the evolution and adaptation of various bacterial strains. The consistent improvement in the bacterial resistance towards drugs and other antibiotics has compelled the researchers to look for new antibacterial agents with improved screening^7^. It is established from the earlier researches that, graphene via its sharp edges can rupture the bacterial cell membranes, thereby making it a promising candidate for bactericidal applications^8–12^. Graphenic structures embedded with inorganic nanoparticles are also well known for their antibacterial performance and low cytotoxicity towards the mammalian cells^13^.

There are many reported studies on various synthesis methods of graphene-silver nanoparticles (AgNPs) and their antimicrobial activity^13–16^. Even though AgNPs are capable of antimicrobial screening, the studies on the adverse effects of nano-silver particles and Ag^+^ ions on human cells as well as on the environment poses a serious health concern^15–18^. Hence, development of alternative materials with comparable antibacterial performance is the need of the hour. Recently, metal oxides are highly sought after for composite preparations in the field of biomedicine primarily due to their high chemical reactivity making them extremely suitable for nanoparticle formation^7,19^. Studies have confirmed that metal oxide nanoparticles considerably decrease the attachment and viability of bacterial agents thereby making them befitting for many antibacterial applications^20–22^. Liu *et al*. reported an increased antimicrobial activity of graphene quantum dot/ZnO nanocomposite under UV photo irradiation due to the enhanced generation of reactive oxygen species (ROS)^21^.

Tin oxide (SnO_2_) is one of the notable metal oxides which possess excellent electrochemical, optical and electronic properties. Recently, Amininezhad *et al*. reported the inactivation efficiency of tin oxide (SnO_2_) nanoparticles for gram-negative *Escherichia coli* and gram-positive *Staphylococcus aureus* under UV and dark conditions^23^. Wu *et al*. investigated the antibacterial activity of graphene-stannous dioxide nanoparticles against *Staphylococcus aureus* and *Pseudomonas aeruginosa* (*P*. *aeruginosa)* using optical density and plate counting methods^12^. It was concluded that the presence of SnO_2_ particles on the graphene framework enhanced the cytotoxicity of the latter because of synergic effect^7,12^.

*P*. *aeruginosa* is a pernicious pathogen that opportunistically affect an immuno-compromised host subjected to medical interventions such as chemotherapy, surgery or any other pre-existing disease^24^. This multi-drug resistant bacterial species is a common cause of nosocomial infections and is also known to alter its phenotype to adapt to the environment^25^. It can even be cultured from distilled water as reported by Favero *et al*. and is resistant towards many disinfectants and bacteriostatic agents^26,27^. It also leads an individual with compromised defense mechanism to develop meningitis, pneumonia, typhilitis, diarrhea etc. The cell envelope present in the bacterium is specially designed to adapt to the environment and resist the intake of synthetic antibacterial agents in to the cell^27^. It is believed that among the gram-negative bacterial strains, *P*. *aeruginosa* possesses the highest attributable mortality^28^. Thus, developing an anti-bacterial agent with high efficacy towards *P*. *aeruginosa* bacterial strains is a challenge and is also very essential for the disinfectant and sanitation industry^29,30^.

Most often, the precursor material used in the studies reported earlier are either graphene/graphite powder or other relatively expensive materials which are commercially available^31^. The predetermined physicochemical characteristics of these graphene domains is disadvantageous because properties of graphene oxide (GO) play an important role in regulating the morphology of metal oxide nanoparticles such as SnO_2_ during the formation of nanocomposite^32^. Production of graphene nanostructures from materials like graphite and its oxides involves procedures which are either expensive or use hazardous chemicals like hydrazine monohydrate^33^. Also, the scissoring of large graphene domains into smaller ones is tedious and time consuming^34^. Barbolina *et al*. has suggested that the antibacterial mechanism and bacterial viability of graphene oxide heavily depends on the precursor as well as on the methods employed in the production of the same before the antibacterial screening^35,36^. Hence, search for an effective precursor that can be developed into a strong antibacterial agent is necessary. In this regard, graphene and metal oxide nanoparticles showcasing relatively high antibacterial properties are worth investigating. However, the quest for a facile and viable means of production of these graphene nanohybrids with metal oxides is often hindered either by herculean protocols or expensive ways of manufacturing. An economically scalable, large-scale route of production of graphene-metal oxide nanohybrids is quite essential to facilitate their usage in variant applications^37^.

Use of renewable resources for the synthesis of nanostructures has been gaining limelight in recent years^38,39^. Yadav *et al*. has recently reported the high antibacterial potential of iron nanoparticles synthesized from *Aloe vera* against various pathogenic bacteria^40–42^. In the current study, we present the synthesis of graphene oxide from the burnt remnants of wood and coconut shell thereby recycling these otherwise waste materials which are rich in activated carbon. A facile and scalable synthesis of graphene oxide nanolayers and nanodots from agro waste materials via a modified Hummers’ method is reported. A simple one step hydrothermal route is adopted for the synthesis of graphene-tin oxide (GTO) nanocomposite. Antibacterial property of the GTO nanocomposites is investigated against *P*. *aeruginosa*, one of the highly resilient bacterial strain known to mankind.

## Results and Discussion

GO obtained by a simple, scalable oxidative treatment of wood charcoal (WC) and coconut shell charcoal (CSC) is code named as WC-HS (WC-Hummers’ treated) and CSC-HS (CSC-Hummers’ treated) respectively. The GTO nanocomposites derived from WC-HS and CSC-HS is further coded as WCT and CSCT respectively. The effective inherent reduction of GO to graphene is facilitated by the use of the one-step hydrothermal treatment with tin (Refer Materials and Methods). Unlike the conventional methods used, absence of hazardous chemical reagents and toxic industrial waste or byproducts during the nanoparticle formation is an added advantage. In addition to this, the adopted method has also achieved the otherwise time-consuming scissoring of large particles into smaller domains.

**Figure 1:**
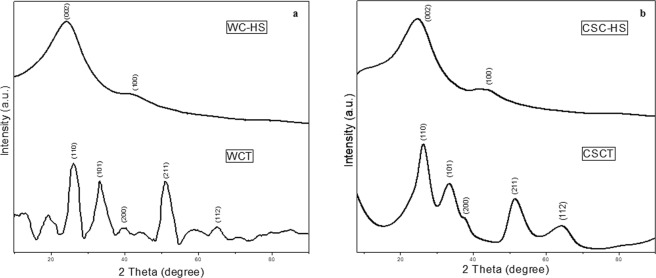
X-ray diffractograms of GO and GTO nanocomposite derived from (**a**) wood charcoal and (**b**) coconut shell charcoal.

X-ray diffractograms of GO and GTO nanocomposites derived from wood charcoal and coconut shell charcoal are given in Fig. 1(a,b) respectively. Chemical modification of the carbon backbone as a result of the oxidative and hydrothermal treatment is evident in the X-ray diffraction (XRD) profiles of GO and GTO nanocomposites in comparison with that of the carbonized precursor materials (Supplementary Information Fig. S1). The signature peaks of GO at ~26° and ~43° arising from (002) and (100) planes respectively are clearly visible in the diffractograms of oxidized samples implying the incorporation of oxygen moieties in the carbon skeleton^43–45^. With the hydrothermal treatment, XRD profiles are modified with the appearance of the distinctive peaks of SnO_2_ suggesting the successful incorporation of tin oxide nanoparticles in the graphene network. In GTO composites, the (002) peak corresponding to graphene domain is seen to be overlapping with the diffraction peak of SnO_2_ from (110) plane. Inherent reduction of graphene oxide during the hydrothermal treatment is apparent from the decreased width along with a slight shift in position of the (002) peak in the composite spectra^46^. In addition to the graphitic peaks, GTO spectra also features peaks at ~26°, ~33°, ~38°, ~51° and ~64° corresponding to (110), (101), (200), (211) and (112) planes of SnO_2_ respectively^47^. From XRD analysis, it is observed that the nanocomposite derived from coconut shell charcoal has distinctively well-defined characteristics in comparison with that obtained from wood charcoal. The presence of oxygen functionalities is also confirmed by Fourier Transform Infrared (FTIR) spectral analysis (Supplementary Information Fig. S2(a,b)) which revealed the presence of stretching vibrations of O-H (~3400 cm^−1^), C=O (~1590 cm^−1^) and C-OH (~1200 cm^−1^) in the oxidized samples. GTO nanocomposites showed the existence of O-H (~3400 cm^−1^), C=C (~1610 cm^−1^), C-H (~1187 cm^−1^), C=O (~1030 cm^−1^) and Sn-O (~600 cm^−1^) stretching vibrations substantiating the successful incorporation of SnO_2_ nanoparticles in the graphene matrix^12,32^.

**Figure 2:**
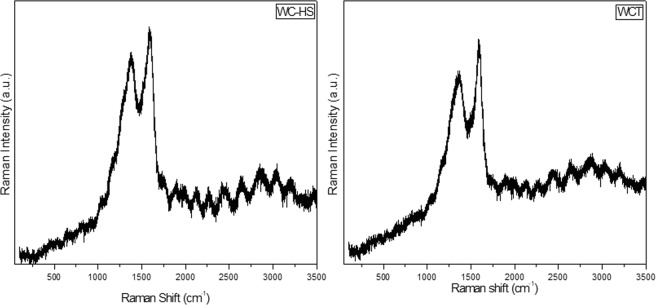
Raman spectra of GO and GTO nanocomposite derived from wood.

**Figure 3:**
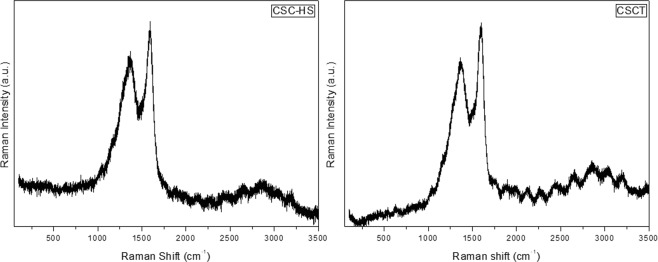
Raman spectra of GO and GTO nanocomposite derived from coconut shell.

Raman spectra of nanocarbon derivatives obtained from carbonized botanical waste materials are presented in Figs 2 and 3 respectively. One could find the existence of defect (D) band (at ~1380 cm^−1^) and graphitic (G) band (at ~1585 cm^−1^) in the synthesized material^48,49^. Raman analysis of the precursors is given in Supplementary Information Fig. S3. The defect (I_D_) to graphitic (I_G_) band intensity ratio is found to be 0.68, 0.92 and 0.93 for WC, WC-HS and WCT respectively indicating the formation of defects or functional groups in the graphene layers. This ratio is calculated to be 0.69, 0.88 and 0.89 for CSC, CSC-HS and CSCT respectively. The higher value of I_D_/I_G_ ratio conventionally indicates the effectiveness of the oxidation process which rises the number of defects^39,49^. The increased intensity of D band is also attributed to the edge effects present in the graphene network of the sample. From Raman analysis, it is evident that the incorporation of SnO_2_ nanoparticles has not caused much change in the graphene skeletal network^50,51^. It can be concluded that the derived nanomaterials mainly consist of sp^2^ carbon with some sp^3^ carbon structures embedded with oxygen functional groups on their surface.

The defects present in the sample are speculated to increase the ROS formation thereby contributing to an increased antibacterial activity of the sample. Moreover, the presence of edges and other induced defects enhances the available surface area for molecular attachments resulting in an elevated toxicity which aids the bacteriostatic and bactericidal performance of the nanostructures^7,52^. Hence, after a relative increase seen in the number of defects via oxidative and hydrothermal treatments, one could expect the rise of antibacterial performance of the respective samples.

**Figure 4:**
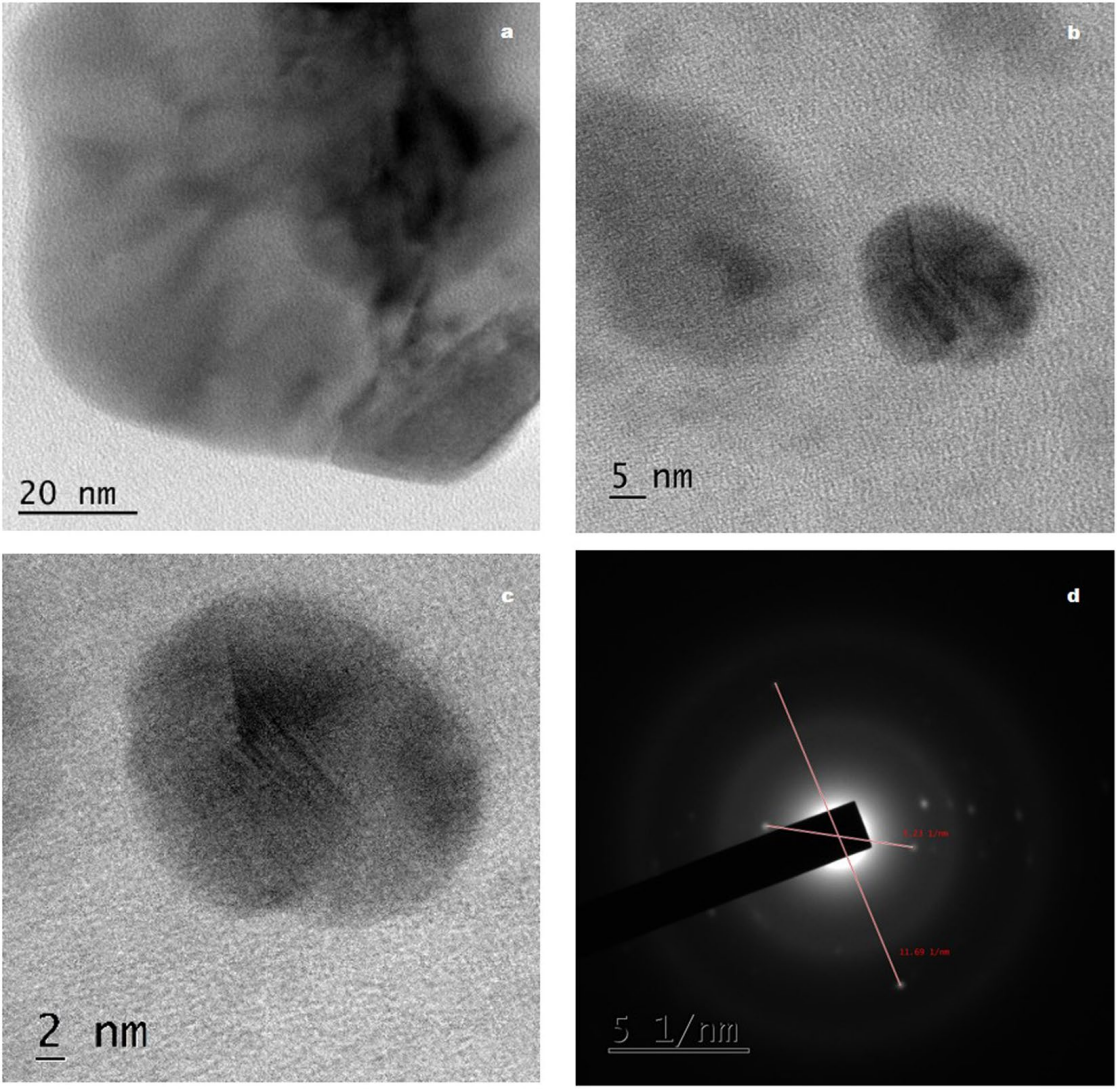
HRTEM images (**a**–**c**) and SAED pattern (**d**) of GO derived from wood.

**Figure 5:**
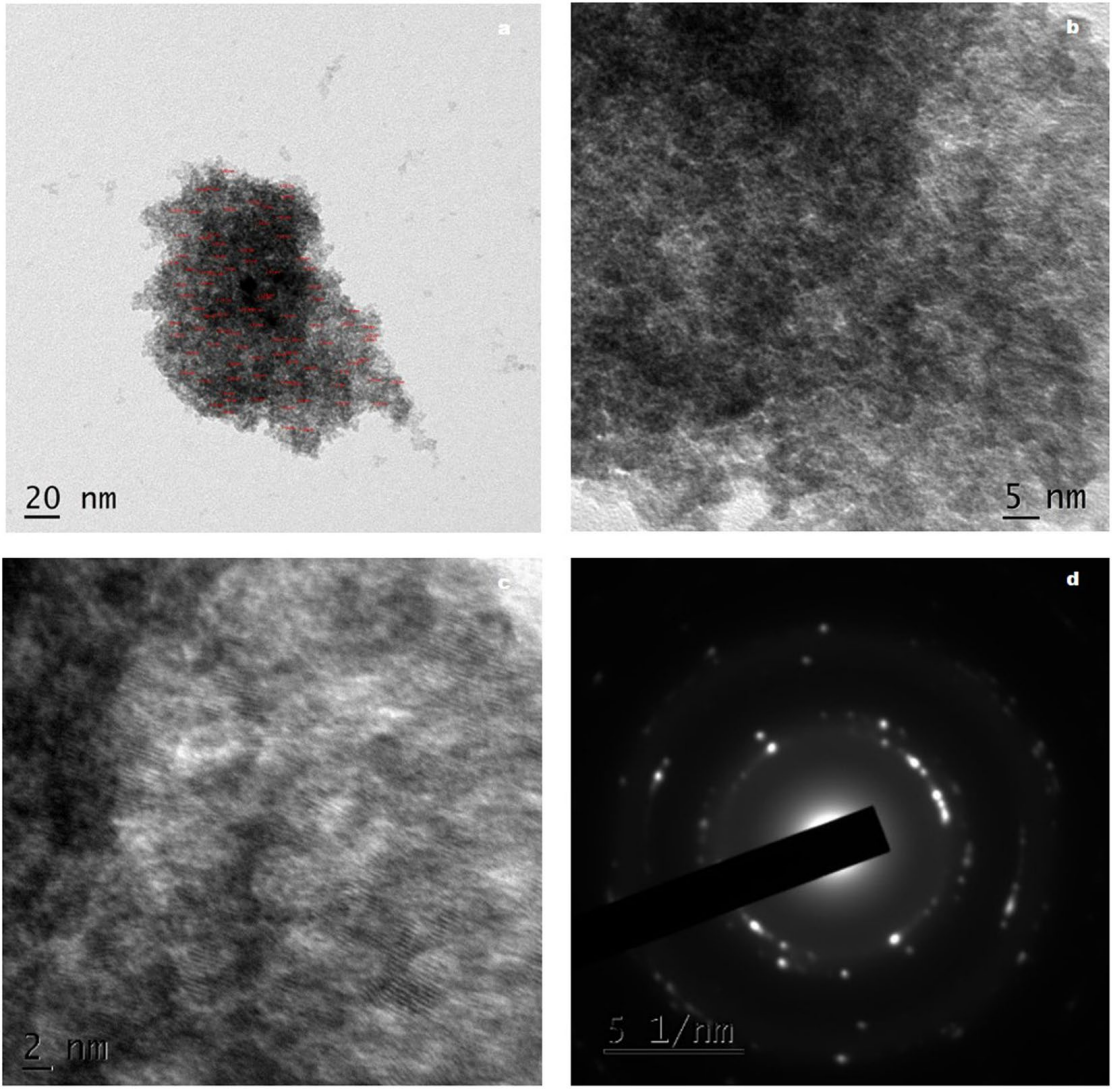
HRTEM images (**a**–**c**) and SAED pattern (**d**) of GTO nanocomposite derived from wood.

High Resolution Transmission Electron Microscope (HRTEM) analysis of WC-HS and WCT are shown in Figs 4 and 5 respectively and that of virgin sample (WS) is presented in Supplementary Information (Fig. S4(a,b)). The larger sheet like morphology of WC is maintained by the oxidized nanostructure. WC-HS images reveal the formation of wrinkled GO nanolayers. In the morphological analysis of WCT, large graphene sheets are seen to be decorated uniformly by SnO_2_ nanoparticles. The even distribution of oxygen functional groups on the graphene sheets facilitates the uniform dispersion of SnO_2_ nanoparticles on the graphene backbone. HRTEM images of WCT show tin oxide nanoparticles with size <3 nm evenly distributed throughout the graphene surface (Supplementary Information Fig. S5). Addition of Sn nanoparticles has resulted in the inherent reduction of GO. It also prevents the aggregation of GO layers which will otherwise affect its antibacterial activity adversely. The change in the crystallinity of GO sample from amorphous to nanocrystalline after the hydrothermal treatment is clearly evident in the selected area electron diffraction (SAED) pattern.

**Figure 6:**
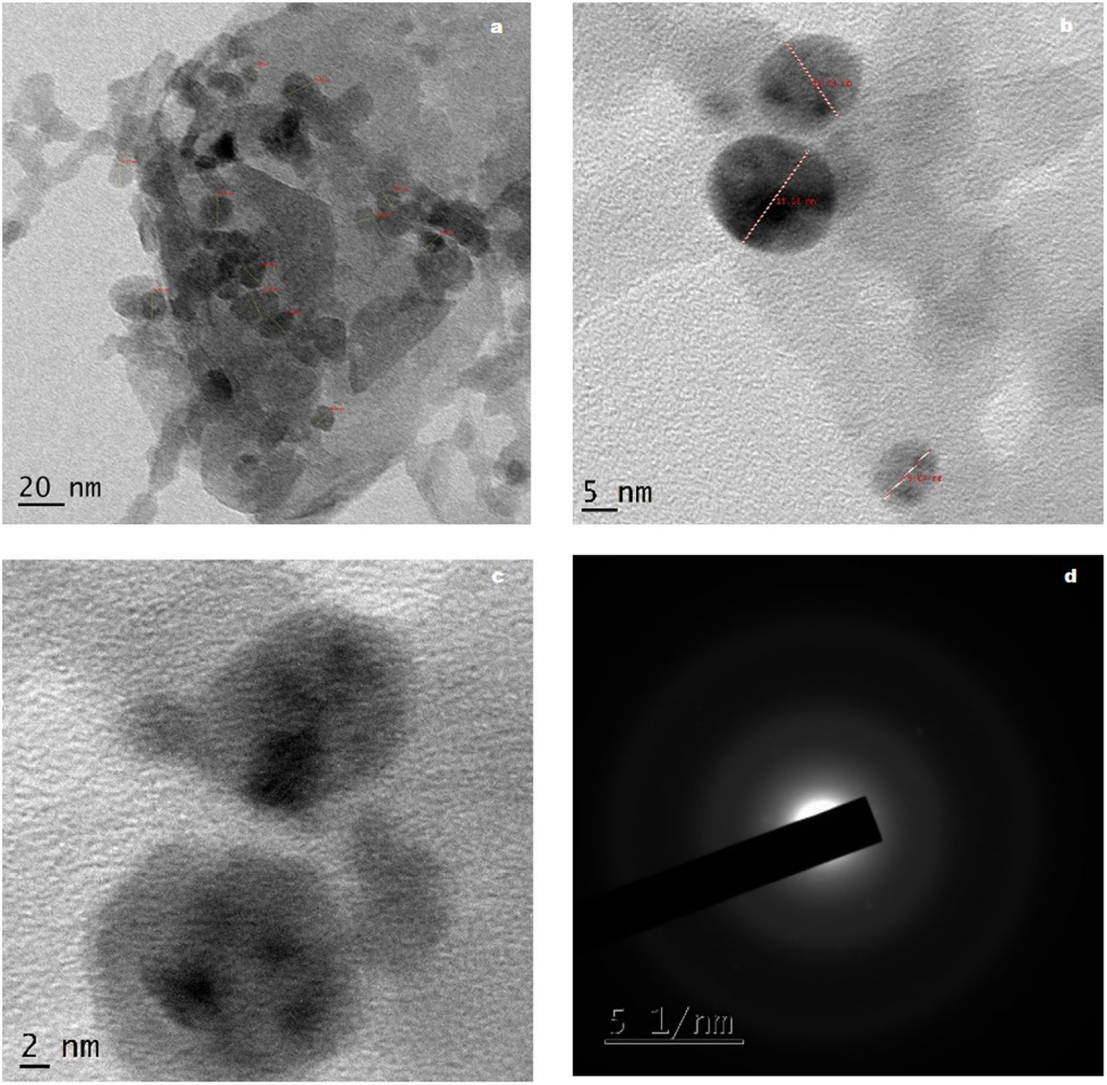
HRTEM images (**a**–**c**) and SAED pattern (**d**) of GO derived from coconut shell.

HRTEM images of the chemically oxidized coconut shell charcoal (CSC-HS) is depicted in Fig. 6. In comparison with the HRTEM images of the virgin sample (Supplementary Information Fig. S6(a,b)), the morphological transformation after the oxidative treatment is evident. Functionalization of the carbon surface has resulted in the exfoliation and effective scissoring of graphene domains along the oxygen groups during sonication. One could observe the formation of a neural network of carbon nanodots embedded in smaller graphene sheet-like background in CSC-HS. The size of the embedded dots ranges from 8–15 nm.

**Figure 7:**
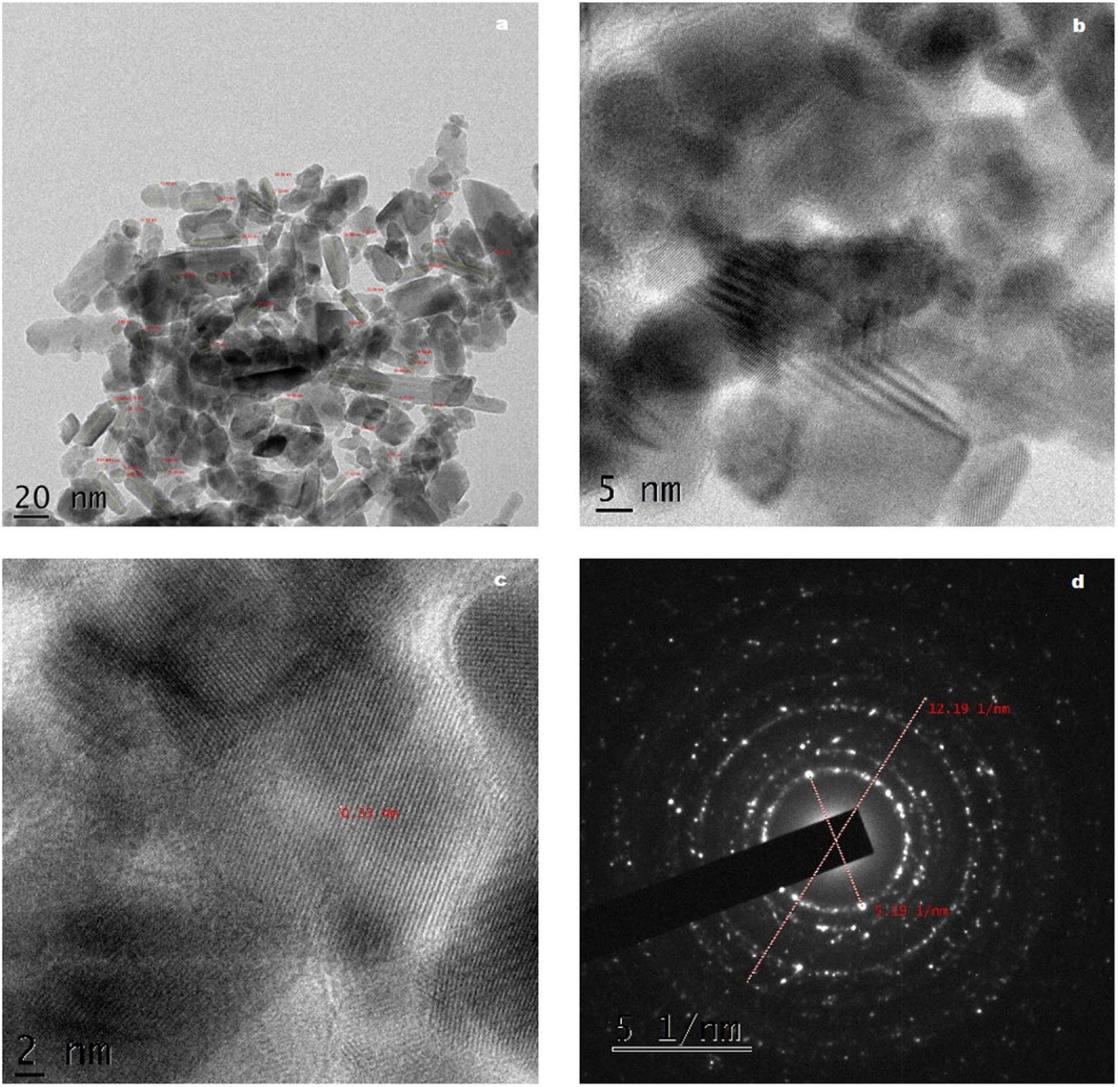
HRTEM images (**a**–**c**) and SAED pattern (**d**) of GTO nanocomposite derived from coconut shell.

HRTEM images of graphene-tin oxide nanocomposite obtained from CSC-HS is depicted in Fig. 7. Destruction of the neural network of carbon dots is apparent from the images. Incorporation of SnO_2_ nanoparticles on the carbon dots framework has resulted in their effective separation. Tin oxide nanoparticles appear to be stacked in the nanocarbon matrix. The lattice fringe width is found to be 0.33 nm which corresponds to (110) plane of SnO_2_. Particle size measured from the HRTEM image evidently falls into two categories: carbon dots with size ranging from 5–11 nm and Sn nanorods of length 20–34 nm. Nanorods are particularly believed to be a better shape among the others in combating the infectious bacteria due to their exposed planes and increased reactivity^53^. Smaller the size of the nanoparticles, higher the surface to volume ratio, which in turn increase the availability of ROS in the sample. ROS plays a pivotal role in enhancing the antibacterial activity by causing membrane damage to the bacterial cells^54^. SAED pattern clearly shows the high crystallinity of the sample with twinning planes belonging to carbon and tin.

Comparing WC and CSC derived nanostructures, a difference in morphology is very evident. Considering the fact that the protocol used for the synthesis of both nanocomposites are the same, this variation in morphology of the two can be ascribed to the structural difference in the precursor materials used. The degree of oxidation of nano-systems is known to be greatly influenced by the morphology of the precursor materials^55^. When WC displayed sheet like carbon domains, CSC possessed relatively smaller polygonal structures. The larger size of the carbon domains present in WC was retained by WC-HS during the oxidative treatments whereas in the case of coconut shell, oxygen group embodiment has resulted in the separation and scissoring of larger particles in to smaller dots. The size and shape of the tin oxide nanostructures adhering to the graphene surface is greatly controlled by the physicochemical traits of GO used in the nanocomposite synthesis^32^. As a result, post-hydrothermal treatment, spherical SnO_2_ nanoparticles are observed in WCT whereas rod-shaped structures are formed in CSCT. Thus, from the structural and morphological analysis, it is suggested that the nanoparticle formation and properties are heavily influenced by the precursor characteristics.

**Figure 8:**
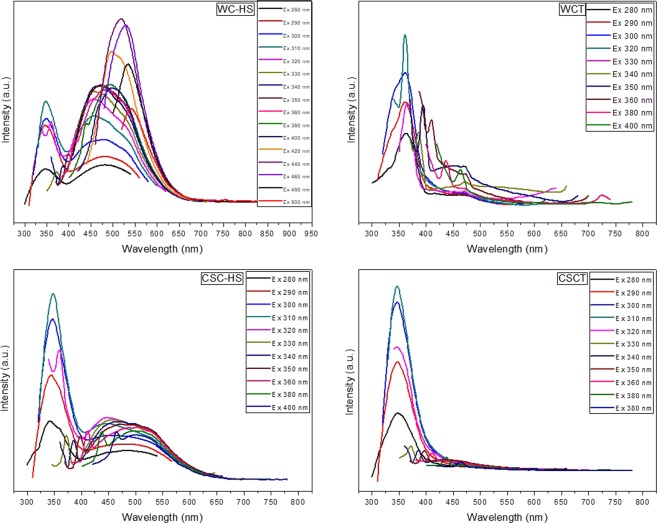
PL spectra of GO and GTO nanocomposite derived from wood and coconut shell.

Photoluminescence (PL) spectrum of the samples are recorded and compared as a function of excitation wavelength. PL spectrum of GO obtained from WC and CSC is presented in Fig. 8 and that of virgin sample is depicted in Supplementary Information Fig. S7. Even though the fluorescence behavior of graphene oxide is widely investigated, the change in fluorescence response of graphene nanostructures after the incorporation of tin oxide nanoparticles are seldom explored. In this study, we report the effect of tin oxide nanoparticles on the luminescence behavior of the graphene oxide samples. Nanostructured WC-HS excited from 300–500 nm in steps of 10 nm exhibits an excitation dependent fluorescent behavior with maximum emission recorded for an excitation of ~310 nm. Development of multiple peaks exhibiting a bathochromic shift could be observed with an increase in excitation wavelength. The emission peak centered at ~350 nm is believed to be originated from the carbogenic core where as the one at ~475 nm belongs to the oxygen functionalities attached during the Hummers’ treatment. Intensities of both the emission peaks exhibit a bell curve pattern with an increase in the excitation wavelength. At ~420 nm, the emission from the carbon core suddenly gains intensity and climbs to a maximum at ~440 nm. The peak intensity is seen to be reducing with a further increase in the excitation wavelength. Fluorescent mechanism of WC-HS can be speculated to be a combinational effect of quantum confinement and oxygen functional groups. With the incorporation of SnO_2_ nanoparticles, a transition of PL characteristics from an excitation dependent to an excitation independent form could be observed. As the excitation changes, the emission intensity was found to be ascending to a maximum at 320 nm and starts decreasing with further increase in the excitation wavelength. The bell curve pattern is still evident in the emission intensities. Absence of the broad satellite peak corresponding to the oxygen functionalities is also noted which supports our claim of a high degree of reduction during the hydrothermal treatment^56^. A fine tuning of the emission peaks from the carbon core is observed with the incorporation of tin nanoparticles. Both quantum size effect and surface effect could be responsible for this excitation independent behavior showcased by GTO nanocomposite^56–58^.

Nanostructured CSC-HS excited from 280 to 400 nm exhibits a fluorescent dependent behavior with the emission maxima of the carbon core centered at ~310 nm. A broad emission from the functional groups could also be seen at a higher wavelength range. With the incorporation of tin nanoparticles, the fluorescence properties changed altogether. From the PL spectrum of CSCT, it is evident that the change in excitation wavelength has no particular influence on the emission characteristics. The removal of oxygen functional groups could also be noted from the absence of the peaks at higher wavelengths. Both GO and GTO nanostructures obtained from WC and CSC showcases similar pattern of emission intensities with change in excitation wavelengths. So, from the PL analysis of the samples, it is deduced that the nanostructures derived from WC and CSC via Hummers’ treatment show an excitation dependent behavior while with the incorporation of tin nanoparticles, PL response becomes independent of the excitation wavelength. Also, the coconut shell derived GTO nanocomposite manifest a better refined excitation independent PL behavior in comparison with those obtained from wood. The synthesized water-soluble nanostructures are found to be highly stable and showed no sign of aggregation even after a storage period of 30 months. They also retained their fluorescence property without any quenching after such extended period of storage.

**Figure 9:**
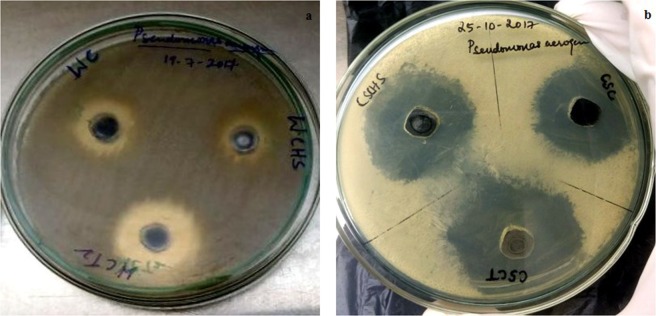
Photographs of the zone of inhibition produced by charcoal, GO and GTO nanocomposite derived from (**a**) wood and (**b**) coconut shell.

The bactericidal test of the synthesized samples (conc. 1 mg/mL) is performed against gram-negative bacteria *P*. *aeruginosa* on nutrient agar plates and the zone of inhibition is measured after incubating for 24 hours. The repeatability of the antibacterial screening of all the samples are also tested for three times. Gram-negative bacterial strain additionally possesses an outer layer of lipopolysaccharides and phospholipids as compared to the gram-positive bacteria and this outermost membrane often acts as a coverage wall for the bacteria thereby rendering it protection from the attack of foreign bodies^7,8,18,59^. At the end of incubation period of the disc diffusion assay with the synthesized nanomaterials, a clearance zone is seen to be developed around the well indicating the antimicrobial activity of the sample. Absolute alcohol is used as the negative control and antibiotic cephalexin is used as the positive control for the study. Figure 9(a,b) shows the zone of inhibition of the precursors, GO and GTO nanocomposites obtained from WC and CSC respectively. It is observed that GTO nanocomposites produced wider inhibition zones compared to their precursor and GO counterparts. The recorded zone of inhibition for WC, WC-HS and WCT are 20 ± 1 mm (number of experimental trials, n = 3) cm, 17 ± 0.8 mm (n = 3) and 27 ± 1 mm (n = 3) respectively. For CSC, CSC-HS and CSCT, the zone of inhibition is measured as 30 ± 1 mm (n = 3), 34 ± 0.8 mm (n = 3) and 38 ± 0.7 mm (n = 3) respectively. No clearance zone is observed in the case of absolute alcohol that is used as the negative control (Supplementary Information Fig. S8). Positive control registered a zone of inhibition of 40 ± 0.9 mm (n = 3). A most commonly used antibiotic drug clarithromycin is also tested as a positive control for comparison and the zone of inhibition is found to be 21 ± 0.7 mm (n = 3) (Supplementary Information Fig. S8). Antibacterial activity of the nanocomposite derived from coconut shell is found to be better than those obtained from WC and is also comparable to that of the positive control cephalexin. All nanostructures obtained after the oxidative and hydrothermal treatment exhibited higher antibacterial potential in comparison with clarithromycin. The measurements are expressed with mean value and standard deviation from three separate experiments (Supplementary Information Fig. S9).

The antibacterial screening is observed to be more in the case of GTO nanohybrids comparing to its virgin and GO counterparts. This can be ascribed to the inclusion of tin oxide nanoparticles together with an increase in number of defects in GTO composites causing an increased surface to volume ratio and more oxidative stress in the bacterial cell walls. The oxidative stress induced in the bacterial membrane helps in incapacitating the inherent anti-oxidant defense mechanism of the bacterial cells^60^. Sharp edges of the graphene nanosheets also aids in the rupturing of the bacterial cell wall making it forego the cell integrity. A considerable increase in the antimicrobial effects with a relative decrease in size of the particles is also reported^60,61^. Hence, the smaller size of nanoparticles formed post-hydrothermal treatment plays a significant role in the enhancement of antibacterial activity of the obtained nanocomposites.

Zone of inhibition measured in this study is by far one of the highest reported. Graphene by itself can help in enhancing the antibacterial performance of the nanocarbon by adsorbing to the bacteria^61^. A slight decrease in the observed antibacterial activity of WC-HS points to the earlier reports that the purity of GO depends on the precursor material used and consequently has a direct relation to its antimicrobial effects. The comparatively less surface area of larger GO domains in WC-HS decreases the interaction sites of the nano-system with bacterial surface leading to a diminished antibacterial activity. In comparison with the nanostructures obtained from wood charcoal, those derived from coconut shell manifests a higher antibacterial activity towards *P*. *aeruginosa*. CSCT showcases 40.7% higher bactericidal capability when compared to WCT. This is a direct consequence of the morphological difference between the two. Nanostructures synthesized from coconut shell charcoal is found to be smaller in size and possess a superior rod like morphology which contributes in enhancing its microbial activity. The increased surface to volume ratio of CSCT nanoparticles significantly increases the surface area available for interaction with the bacterial membrane leading to a higher bactericidal efficacy. Also, larger particles tend to get adsorbed on the bacterial surface whereas the smaller ones get absorbed in to the bacterial cell resulting in internal damage and alterations in the cell activity^62^. Hence, CSC nanostructures possessing smaller carbon domains are more effective in eradicating the bacterial strains. The increase in number of defects in the graphene backbone, release of toxic ions in to the bacterial cell and the diminution of surface negative charge after nanocomposite formation might have also benefited the cause^12,13^. GTO nanohybrid appears to combine the antibacterial properties of both GO and SnO_2_ particles, effectively enhancing the antibacterial effect of both the base materials^11^.

**Figure 10:**
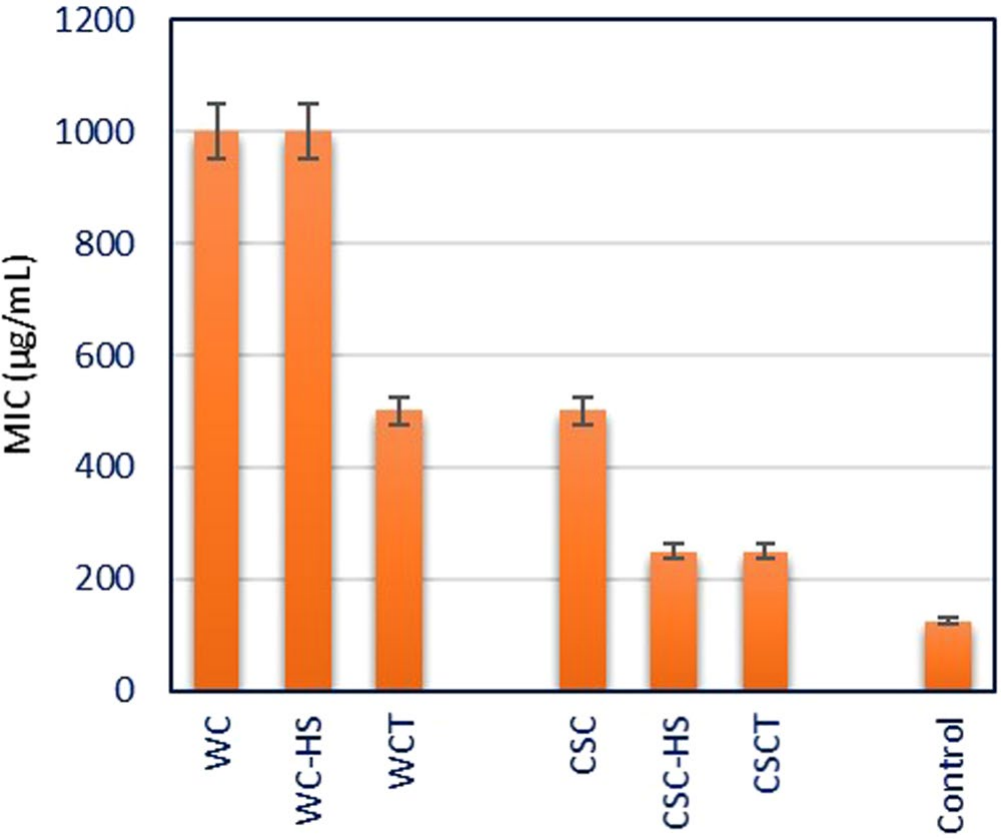
MIC assay of synthesized nanostructures against *P*. *aeruginosa*.

Minimum inhibitory concentration (MIC) of the synthesized nanostructures against *P*. *aeruginosa* is determined by microdilution plate method with resazurin^63^ (Supplementary Information Fig. S10). The inoculated plates are prepared in triplicate and incubated for 20 hours at 37 °C. Antibiotic cephalexin is used as the control. Resazurin is a purple colored dye that turns pink in the presence of viable bacterial cells. The minimum concentration of the sample required to prevent the bacterial growth is taken as the MIC. MIC assay of the synthesized nanostructures (Fig. 10) revealed that GTO nanocomposites has lower MIC values than the respective precursor and GO nanostructures. Nanoparticles synthesized from CSC exhibited higher antibacterial potential whereas WC nanostructures displayed weaker growth inhibition of *P*. *aeruginosa*. Among the synthesized nanostructures, the least MIC value is recorded for CSCT (250 μg/mL) implying the efficacy of the coconut shell derived GTO nanocomposite.

From the disc diffusion assay and MIC analysis of WC and CSC derivatives, it is evident that GTO nanocomposites have better antibacterial efficacy in comparison with the respective precursor and GO counterparts. Zone of inhibition measured in the case of GTO nanocomposite from CSC is comparable to that of the standard antibiotics used. This is in turn corroborated by the low MIC value of CSCT in MIC assay. Thus, GTO nanocomposite derived from coconut shell seems to be promising for various antimicrobial applications.

## Conclusion

In summary, an efficient and easily scalable strategy for the economic large-scale synthesis of water-soluble graphene oxide and graphene-tin oxide nanocomposites from agricultural green carbon wastage is demonstrated. A uniform distribution of tin oxide nanoparticles in the graphene matrix is observed. Morphological difference between WCT and CSCT nanocomposites produced using identical chemical protocol indicates that the initial precursor material used for the graphene oxide synthesis plays a pivotal role in the nanocomposite formation. All the synthesized nanostructures exhibited high antibacterial activity and among them, GTO nanocomposites showcased a marked raise in antibacterial screening. Zone of inhibition exhibited by GTO nanocomposites derived from wood charcoal and coconut shell charcoal against *Pseudomonas aeruginosa* are 27 ± 1 mm and 38 ± 0.7 mm respectively. This measured antibacterial activity is one of the highest reported of its kind. MIC assay has revealed that CSCT nanocomposite could impede the bacterial growth at lower concentrations than the other synthesized nanostructures. The rod-shaped morphology and well-defined structural characteristics of GTO nanocomposite derived from coconut shell has made them more efficient antimicrobial agents in comparison with WC nanostructures. The effective increase in number of defects as a consequence of the chemical treatments employed has contributed strongly towards the enhancement of antibacterial performance of the nanocomposites. The upgradation of fluorescence behavior to an excitation independent response after the hydrothermal treatment makes GTO nanocomposite a potential benign candidate for biomedical applications. The nanomaterials synthesized are highly stable and showed no sign of aggregation or photo quenching even after extended storage periods. It can be concluded that, CSC derived GTO nanocomposite holds great promise as an antimicrobial agent in various applications like food packaging, sanitary agent manufacturing, sewage treatments, etc. and needs to be investigated further.

## Materials and Methods

### Synthesis of GTO nanocomposite from wood and coconut shell charcoal

The formation of GTO nanocomposites was done in three steps. The remnants obtained after the carbonization of wood (~1 kg for ~320 g WC) and coconut shell (~1 kg for 300 g CSC) were grounded and washed thoroughly as the first step. In the second step, dried samples were subjected to oxidative treatment by using a modified Hummers’ method described as follows. 2 g of the respective precursor along with 2 g of NaNO_3_ was dispersed in 26 ml of H_2_SO_4_. 6 g of KMnO_4_ was added slowly in steps to the above mixture. The system was kept in an ice bath to prevent overheating. The mixture was stirred for 48 hours in order to facilitate complete oxidation. The reaction was terminated by adding 500 ml 5 °C distilled water and 5 ml 30% H_2_O_2_ in order to facilitate the exfoliation of the graphitic domains. The mixture was washed repeatedly with dil. HCl, ethanol and distilled water to get rid of the unoxidized reactants and metal ions. It was further subjected to dialysis for the removal of any leftover acid traces. Once a neutral pH was attained, the samples were sonicated and dried under vacuum to obtain WC-HS (~1.2 g) and CSC-HS (~1.7 g). Third step comprised of a one pot hydrothermal treatment of WC-HS and CSC-HS to obtain GTO nanocomposites and is described as follows. 0.05 g of GO is dissolved in 25 mL ethanol via sonication to ensure the exfoliation of the graphitic domains. At the same time, 2.25 g of SnCl_2_.2H_2_O is dissolved in 25 mL ethanol. Both the assortments were then transferred to a beaker and sonicated for 90 minutes to ensure thorough mixing of the two systems. Sn^2+^ ions get anchored on to the GO sheets during this step. The resultant mixture was heated at 150 °C for 24 hours in a sealed high-pressure Teflon lined autoclave. In this phase, GO undergoes gradual inherent reduction whereas tin nanoparticles get oxidized simultaneously and gets deposited on the graphene surface. The mixture was washed repeatedly with distilled water to eliminate the unwanted chloride ions. The resultant product was then dried in vacuum at 60 °C to obtain GTO nanocomposites WCT (~0.4 g) and CSCT (~1.2 g). The chemicals used were of analytical grade and was purchased from Sigma-Aldrich.

### Antibacterial Studies of the Nanostructures

Antibacterial studies of the samples were carried out against gram-negative bacterial strain of *P*. *aeruginosa* using disc diffusion assay. Sterile Muller-Hinton agar was prepared by autoclaving at 121 °C for about 20 minutes and poured into sterile petri plates under LAF. The plates were allowed to solidify and 100 µL of bacterial suspension *P*. *aeruginosa* (0.5 OD) was spread over the nutrient agar using sterile cotton swabs. Thereafter 3 wells (7 mm diameter) were made on the nutrient agar using a sterile cork borer. 0.01 g of the synthesized sample was dissolved in 10 mL of absolute ethanol. 100 µL of this sample was loaded into each well. The plates were then wrapped using a cling film and incubated at 37 °C for 24 hours. Zone of inhibition produced by the nanostructures was then measured using Antibiotic Zonescale supplied by HiMedia Laboratories Pvt. Ltd. Antibiotic cephalexin and clarithromycin (10 μg/disc) were used as positive controls. Negative control was absolute alcohol without any sample. The experiment was performed in triplicate to minimize the experimental errors.

MIC assay of the precursors, GO and GTO nanocomposite against *P*. *aeruginosa* was carried out using 96 well micro-titer plate method^63^. 100 μL of sterilized Luria Bertani broth was dispensed to each well of the titer plate. 2 mg/mL solution of each sample was prepared in DMSO (Dimethyl Sulfoxide). 100 μL of the sample solution was added to a row of the titer plate and serial dilutions were carried out thereafter. The obtained concentration range was from 1000 to 7.8 μg/mL. 10 µL of bacterial suspension (10^6^ CFU/mL) along with 30 µL of 0.1% of resazurin were added to the sample wells. Antibiotic cephalexin was used as the control. A column of wells dispensed with resazurin was taken as color blank. An adjacent column with both resazurin and bacterial suspension was taken as culture control. The plates were prepared in triplicates and the inoculated plates was incubated at 37 °C for 20 hours.

### Characterization

Structural and morphological properties of the samples were studied using various characterization techniques. X-ray diffraction measurements were carried out using a a Bruker AXS D8 Advance X-ray diffractometer. Functional groups were identified using a Shimadzu FT-IR-8400 spectrometer and a RF-5301 PC, Shimadzu fluorescence spectrometer was used to obtain PL spectra of the samples. Raman analysis was carried out using a Horiba LABAM-HR spectrometer at 514 nm. High resolution transmission electron microscope (HRTEM) images of the samples were obtained using a JEM-2100 (JOEL) model system.

## Supplementary information

Supplementary Information
