# Supplementary material for: Facile synthesis of graphene-tin oxide nanocomposite derived from agricultural waste for enhanced antibacterial activity against Pseudomonas aeruginosa
Source: Sci Rep. 2019 Mar 12;9:4170. doi: 10.1038/s41598-019-40916-9 (PMC6414503; doi:10.1038/s41598-019-40916-9)
Supplement: Supplementary file 1 — Supplementary Information [file 41598_2019_40916_MOESM1_ESM.docx]

**Facile synthesis of graphene-tin oxide nanocomposite derived from agricultural waste for enhanced antibacterial activity against Pseudomonas aeruginosa**

Anu N Mohan^1*^, Manoj B^1^, Sandhya Panicker^2^

^1^Materials Science Research Laboratory, Department of Physics and Electronics, CHRIST (Deemed to be University), Bengaluru-560029, Karnataka, India.

^2^Department of Botany, St. Joseph’s College, Post Graduate and Research Center, Langford Road, Bengaluru-560027, Karnataka, India.

^*^Email: [anunmohan@gmail.com](mailto:anunmohan@gmail.com)

**SUPPLEMENTARY INFORMATION**

**Fig. S1: X-ray diffractogram of WC and CSC**

**Fig. S2: FTIR spectra of (a) WC, WC-HS, WCT and (b) CSC, CSC-HS, CSCT**

[FTIR spectrum of Hummers’ treated samples clearly indicate the incorporation of oxygen functional groups in the carbon backbone after the oxidative treatment. A slight red shift is observed in the case of GTO composites and it is attributed to the electron donating effect.]

**a**

**b**

**Fig. S3: Raman spectra of WC and CSC**


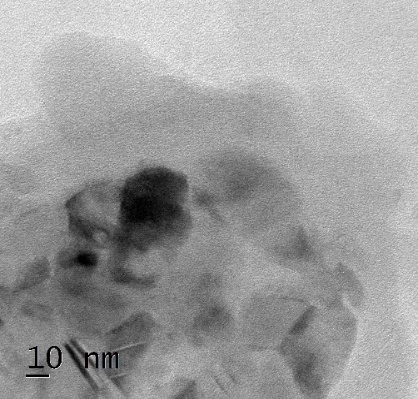

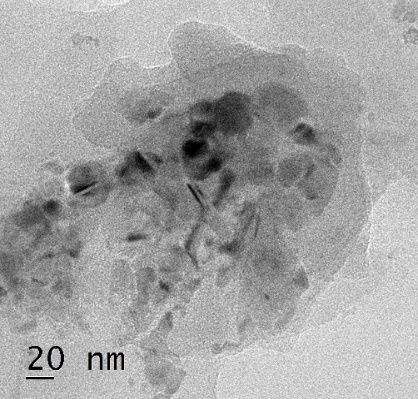


a

b

**Fig. S4: (a-b) HRTEM images of carbonized wood**

**Fig. S5: Particle size histogram of WCT**

[The particle size distribution (formulated from the measurement of 100 particles) of graphene-tin oxide nanocomposite indicates that the particles are uniform sized and are distributed evenly on the graphene surface. It is also observed that the majority of particles falls in the range of 1.25-1.75 nm.]


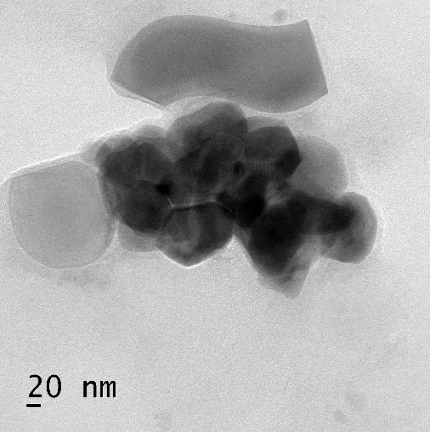

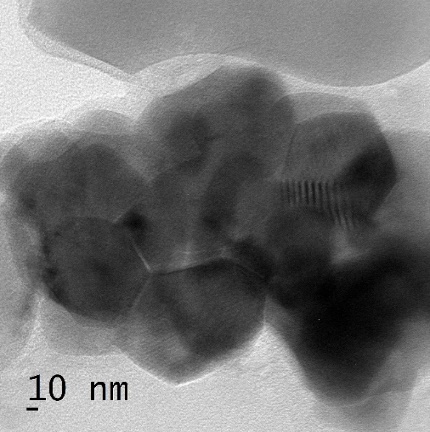


a

b

**Fig. S6: (a-b) HRTEM images of carbonized coconut shell**

**Fig. S7: PL spectra of WC and CSC**

[PL spectra of the carbonized samples shows the characteristic emission peak arising from the carbon core.]

[


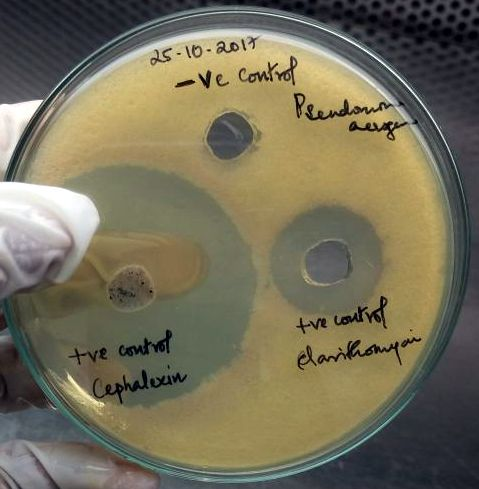


**Fig. S8: Photographs of the controls used in the disc diffusion assay of the nanostructures against P. aeruginosa**

**Fig. S9: Photographs of the replicates of disc diffusion assay of the nanostructures against P. aeruginosa**

[Experiment was performed in triplicate]


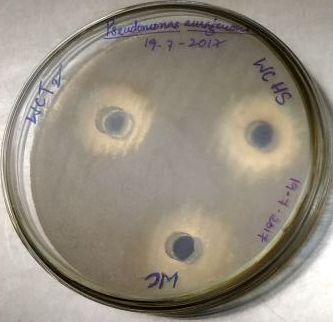

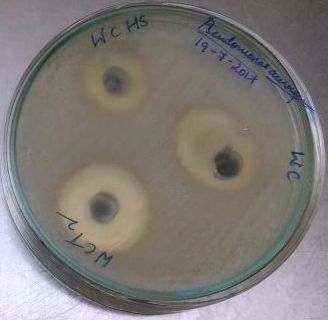

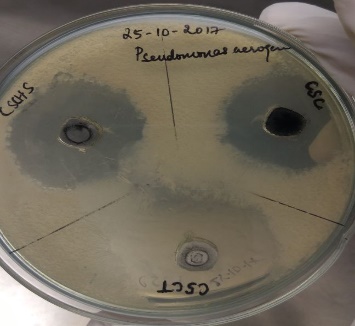

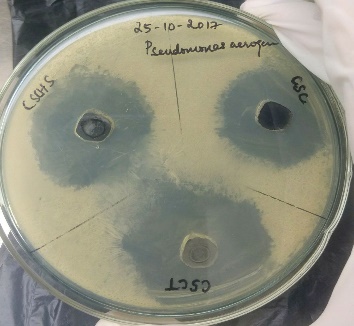

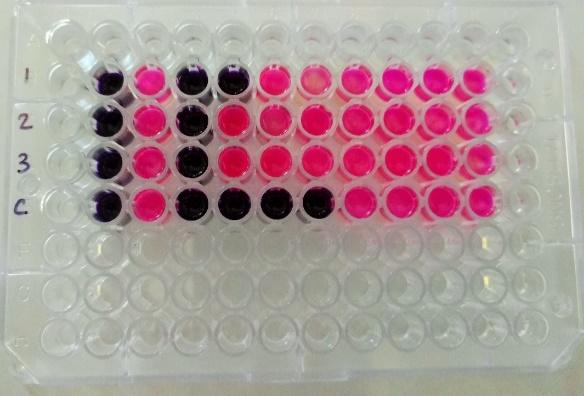

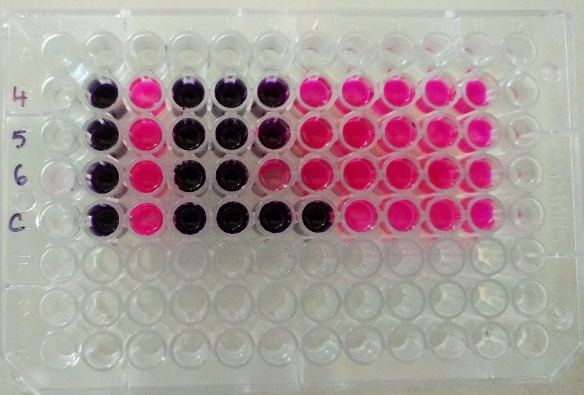


**Fig. S10: MIC assay of the synthesized nanostructures against P. aeruginosa**

**1-WCT; 2-WC-HS; 3-WC; 4-CSCT; 5-CSC-HS; 6-CSC; C-Control**

[Experiment was performed in triplicate]
